# Negative Legal Emotion and Prosocial Behavior: A Moderated Mediation Model of Peer Attachment and Social Exclusion in a Chinese Undergraduate Sample

**DOI:** 10.3390/bs16040579

**Published:** 2026-04-11

**Authors:** Yanbing Xu, Shuhui Xu

**Affiliations:** 1 Department of Student Affairs, Wenzhou University, Wenzhou 325035, China; 2 Department of Psychology, Wenzhou University, Wenzhou 325035, China

**Keywords:** negative legal emotion, prosocial behavior, peer attachment, social exclusion, moderated mediation model

## Abstract

**Introduction**: Negative legal emotion refers to the affective component of negative orientations toward legal norms, institutions, and procedures. It is closely related to, but not synonymous with, the broader construct of legal cynicism, which more often emphasizes generalized skepticism toward the fairness, legitimacy, and effectiveness of the law. This study examined the association between negative legal emotion and prosocial behavior among university students, with peer attachment as a mediator and social exclusion as a moderator. **Method**: A total of 404 undergraduates from mainland Chinese universities were included in the final analysis after predefined data screening, including attention-check items and response-time cutoffs. Participants completed an online survey assessing negative legal emotion, prosocial behavior, peer attachment, and social exclusion. Descriptive statistics, correlation analyses, and Hayes’s PROCESS macro (Model 7) were used to test the moderated mediation model, controlling for gender, grade, and parental educational attainment. **Results**: Negative legal emotion was negatively associated with prosocial behavior. Peer attachment statistically accounted for this association, such that higher negative legal emotion was associated with lower peer attachment, which was in turn associated with lower prosocial behavior. Social exclusion moderated the first stage of the indirect pathway: the negative association between negative legal emotion and peer attachment was weaker at higher levels of social exclusion. Accordingly, the indirect association between negative legal emotion and prosocial behavior via peer attachment also varied across levels of social exclusion. **Conclusions**: The findings suggest that peer attachment is an important relational correlate linking negative legal emotion with prosocial behavior and that social exclusion is associated with variation in the strength of this indirect pathway. These results extend research on legal socialization and contribute to understanding prosocial behavior among university students.

## 1. Introduction

### 1.1. Negative Legal Emotion and Prosocial Behavior

The theory of emotional social structure posits that emotional experiences are embedded in social contexts, interpersonal relationships, and broader cultural systems (Fu, 2016; Rehman et al., 2025). Within the three-dimensional dynamic model of legal socialization, legal socialization is conceptualized as a developmental process through which individuals acquire, internalize, and enact legal norms through interconnected cognitive, emotional, and motivational mechanisms (Xu, 2026). In this framework, legal cognition refers to individuals’ knowledge, interpretations, and reasoning about legal rules, values, and authorities; legal emotions refer to subjective affective responses elicited by legal stimuli, including legal norms, institutional symbols, procedural interactions, and perceived fairness of outcomes; and legal motivation refers to the internal drive to comply with, uphold, and use the law (Xu, 2026). Thus, legal emotion is conceptually distinct from legal cognition and legal motivation, even though it is shaped by cognitive appraisal and may guide motivational orientation.

More specifically, the present study focuses on negative legal emotion, that is, adverse affective responses to the spirit of the law or its institutional operation. According to Xu (2026), negative legal emotions become salient when legal outcomes or legal practices are perceived as violating expectations of fairness, dignity, or moral order, and may be expressed in forms such as disappointment, contempt, and aversion. In this sense, negative legal emotion is related to, but not identical with, legal cynicism. In the criminological and legal socialization literature, legal cynicism is typically defined as a generalized skepticism toward the fairness, legitimacy, and effectiveness of the law and legal authorities (Sampson & Bartusch, 1998; Gifford & Reisig, 2019; Trinkner & Cohn, 2014). By contrast, negative legal emotion emphasizes the affective experience accompanying such evaluations. Accordingly, the present study does not simply relabel legal cynicism. Rather, it examines the affect-laden negative orientation toward law that may coexist with, and partly arise from, broader skeptical evaluations of legal institutions.

Prosocial behavior is not a unitary phenomenon but a multidimensional construct that includes helping, sharing, cooperating, comforting, and other-oriented responding. In the present study, prosocial behavior is treated as a broader tendency toward socially supportive and other-oriented action. Theoretically and empirically, negative legal emotion is likely to be negatively associated with prosocial behavior. Individuals who hold more negative affective orientations toward law and legal authority may be less likely to regard legal norms as morally meaningful or socially binding, and may also show lower trust in the normative order that supports cooperative behavior (Sampson & Bartusch, 1998; Fagan & Tyler, 2005; Gifford & Reisig, 2019; Kaiser & Reisig, 2019; Nivette et al., 2020). Within the triadic model, legal emotion functions as a psychological bridge linking cognitive evaluations of law with motivational and behavioral tendencies (Xu, 2026). Thus, when legal–emotional responses are characterized by disappointment, contempt, or aversion, individuals may be less likely to form positive affective identification with the law or to maintain strong prosocial orientations. At the emotional level, negative legal emotion may also be accompanied by greater attention to grievance, unfairness, or interpersonal threat, which may correspond to lower other-orientation and weaker helping tendencies (Holloway et al., 1977; Carlo & Randall, 2002). At the relational level, negative emotional orientations toward law may be associated with weaker affective identification with institutions and groups, thereby weakening the social and relational foundations that often support prosocial behavior (Fu, 2016; Xu, 2026). Taken together, these theoretical and empirical considerations suggest that negative legal emotion is negatively associated with prosocial behavior and therefore provides a meaningful starting point for the present study.

### 1.2. The Mediating Role of Peer Attachment

Likewise, peer attachment is a multifaceted relational construct involving trust, communication, emotional closeness, and perceived security in peer relationships. Attachment theory suggests that attachment reflects relatively stable affective bonds with significant others and that internal working models are closely related to emotional regulation, trust, and social behavior (Bowlby, 1969, 1980; Mikulincer & Shaver, 2019). Although peer attachment has often been emphasized in adolescence, peer relationships remain developmentally salient in young adulthood, especially during the transition to university. For university students, friendship quality and peer ties are closely associated with belonging, adjustment, loneliness, and mental health (Buote et al., 2007; Swenson et al., 2008; Maunder, 2018; Calderon Leon et al., 2024; Song et al., 2022). Secure peer attachment is also associated with higher prosocial behavior, whereas insecure or disrupted attachment is associated with externalizing tendencies and aggression (Rajchert, 2015; Peng et al., 2021).

Focusing on peer attachment is therefore theoretically appropriate in the present study. In university settings, peers constitute a highly salient microsocial context that provides emotional support, social feedback, and a concrete sense of belonging (Calderon Leon et al., 2024; Maunder, 2018; Swenson et al., 2008). Family relationships and broader social identification are also important, but peer attachment is especially relevant here because it captures the proximal affiliation context in which university students negotiate trust, inclusion, and everyday social behavior.

Framing peer attachment as a mediator between negative legal emotion and prosocial behavior provides a relational account of how legal–emotional orientations may be linked to social functioning. Within the three-dimensional dynamic model of legal socialization, legal emotion occupies a bridging position between legal cognition and legal motivation and is embedded in broader relational contexts (Xu, 2026). From this perspective, negative legal emotion is not assumed to target peers directly. Rather, it may be associated with weaker generalized trust, lower normative endorsement, and reduced social investment in close relationships (Fu, 2016; Sampson & Bartusch, 1998; Xu, 2026). In university students, such broader relational changes may be especially visible in peer attachment because peer ties are among the most salient and accessible sources of affiliation in emerging adulthood (Buote et al., 2007; Calderon Leon et al., 2024; Maunder, 2018).

Peer attachment is, in turn, theoretically relevant to prosocial behavior through several related psychological processes. From an attachment perspective, more secure attachment is associated with stronger relational security, better emotion regulation, and greater trust in close others, all of which are conducive to empathic concern and other-oriented responding (Bowlby, 1969, 1980; Mikulincer & Shaver, 2019; Shaver et al., 2019). Attachment research has also suggested that secure relational bonds are linked to caregiving tendencies and broader prosocial orientation, whereas insecurity is more often associated with defensive responding and reduced social openness (Gross et al., 2017; Shaver et al., 2019). In peer contexts specifically, stronger attachment may be associated with greater belonging, reciprocity, and willingness to invest in cooperative relationships, which are themselves closely related to prosocial behavior (Laible et al., 2000; Rajchert, 2015). Thus, lower peer attachment may correspond to weaker relational security and weaker other-oriented engagement, both of which are associated with lower prosocial orientation and behavior. Prior research also suggests that attachment quality may statistically account for associations between emotional or attitudinal variables and behavioral outcomes (Mota & Matos, 2013; Murphy et al., 2017; Peng et al., 2021). Accordingly, positioning peer attachment as a mediator in the association between negative legal emotion and prosocial behavior is grounded in developmental theory, attachment-based accounts of prosociality, and evidence on peer relations in young adulthood.

### 1.3. The Moderating Role of Social Exclusion

Social exclusion, defined as experiences of being ignored, rejected, or excluded by others or by a group, constitutes a salient interpersonal contextual variable (Du & Xia, 2008; Williams, 2007). Empirical evidence indicates that social exclusion can increase negative affect, impair self-regulation and cognitive functioning, and often reduce engagement in prosocial behavior (Baumeister et al., 2002; Twenge et al., 2007). From a social exchange perspective, when individuals perceive themselves as excluded, they may adopt self-protective motives and reduce investments in others, prioritizing their own well-being (Blau, 1964; Cuadrado et al., 2016; Takhsha et al., 2020).

At the same time, the consequences of social exclusion are not uniformly negative. According to the multimotive model of interpersonal rejection, social exclusion can evoke multiple, potentially competing motives, including prosocial, antisocial, and socially avoidant responses (Smart Richman & Leary, 2009). In particular, when excluded individuals perceive a possibility of relational repair, value the relationship, or remain motivated to regain social acceptance, exclusion may foster affiliative and relationship-promoting behavior as a means of restoring belonging. In contrast, when exclusion is perceived as unfair, chronic, or unlikely to be repaired, it may instead elicit hostility, withdrawal, or reduced prosociality (Smart Richman & Leary, 2009).

Consistent with this perspective, prior empirical findings are mixed. On the one hand, exclusion can amplify negative reactions and suppress prosocial behavior; on the other hand, it can trigger stronger reconnection motives, prompting excluded individuals to engage in compliant or affiliative behaviors to restore social ties (Maner et al., 2007; Williams et al., 2000). Thus, social exclusion should not be conceptualized as exerting a uniformly buffering or uniformly aggravating influence. Rather, its moderating role is likely to be conditional and context-dependent.

Accordingly, in the present study, social exclusion is proposed to moderate the first stage of the mediated model, namely the association between negative legal emotion and peer attachment. Specifically, higher levels of social exclusion may strengthen the disruptive effect of negative legal emotion on peer attachment when exclusion primarily activates defensive, distrustful, or withdrawal-oriented responses. However, higher levels of exclusion may also attenuate this negative association when exclusion activates compensatory affiliation motives and increases the tendency to seek reconnection through peer relationships. Therefore, the moderating effect of social exclusion is treated as exploratory in directional terms: Social exclusion may either exacerbate or buffer the negative association between negative legal emotion and peer attachment, depending on the dominant motivational response elicited by the exclusion experience.

### 1.4. Current Study and Hypotheses

Grounded in emotional socialization, attachment theory, and the three-dimensional dynamic model of legal socialization, the present study examines how negative legal emotion is associated with prosocial behavior among university students, with particular attention to peer attachment and social exclusion. Although prior research has documented associations among legal cynicism or negative legal orientations, peer relations, prosocial behavior, and social exclusion, these lines of work have typically developed in parallel rather than within a single integrative framework. As a result, three issues remain insufficiently specified in the existing literature: whether institution-related negative emotion is associated with everyday interpersonal behavior, whether peer attachment may statistically account for this association, and whether this relational pathway varies across different levels of social exclusion.

Accordingly, the present study is organized around three connected features. First, it focuses on negative legal emotion as the affective dimension of negative orientations toward legal norms, institutions, and procedures, rather than treating broader cognitive–evaluative legal attitudes as the sole focus. Second, it examines peer attachment as a proximal relational pathway through which institution-related emotion may be associated with everyday social behavior. Third, it incorporates social exclusion as a contextual condition that may be associated with variation in this pathway (Fu, 2016; Sampson & Bartusch, 1998; Xu, 2026).

This framework assumes that legal–emotional orientations may be linked to interpersonal functioning through relational processes. Negative legal emotion may be associated with weaker generalized trust, lower normative endorsement, and reduced social investment, which may be reflected in close peer relationships during young adulthood (Fu, 2016; Sampson & Bartusch, 1998; Gifford & Reisig, 2019; Nivette et al., 2020; Xu, 2026). Peer attachment, in turn, represents a developmentally salient relational context for university students and is closely associated with belonging, trust, and prosocial orientation (Buote et al., 2007; Laible et al., 2000; Maunder, 2018; Rajchert, 2015). Social exclusion may further shape this pathway because exclusion has been linked to both maladaptive interpersonal tendencies and compensatory affiliation motives, depending on how the exclusion experience is construed (Smart Richman & Leary, 2009; Maner et al., 2007).

Based on this framework, the present study tested the following hypotheses using a moderated mediation model with bootstrap resampling while controlling for gender, grade, and parental educational attainment:

**H1.**  *Negative legal emotion will be negatively associated with prosocial behavior.*

**H2.**  *Peer attachment will statistically account for the association between negative legal emotion and prosocial behavior.*

**H3.**  *Social exclusion will moderate the association between negative legal emotion and peer attachment; however, given the potentially competing consequences of social exclusion, the direction of this moderating effect is treated as exploratory.*

**Figure 1:**
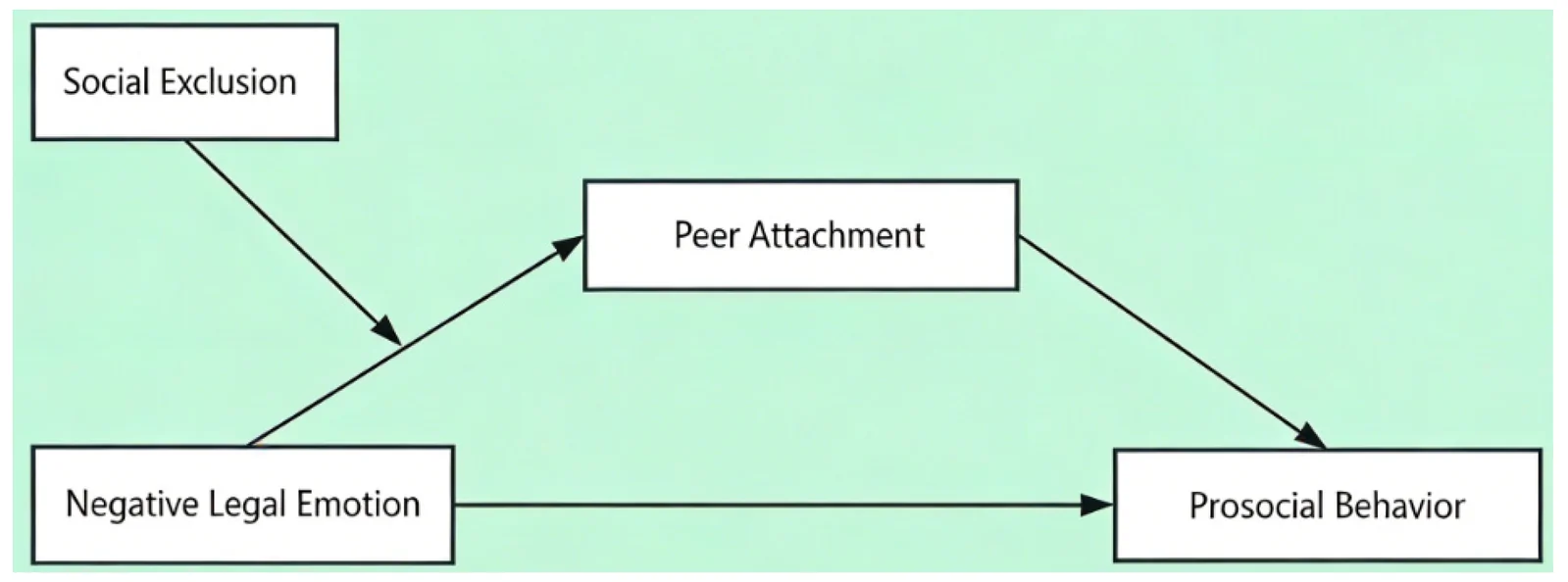
Hypothesized model.

**H4.**  *The indirect association between negative legal emotion and prosocial behavior via peer attachment will vary across levels of social exclusion, with the direction of this moderated mediation effect treated as exploratory. See Figure 1.*

## 2. Methods

### 2.1. Participants

The present study used a questionnaire survey administered to undergraduate students from mainland Chinese universities. Data were collected online via the Wenjuanxing platform and completed in class under group administration by a principal investigator with psychology training. A total of 460 questionnaires were returned.

Data screening was conducted using a predefined set of quality-control criteria. First, based on the completion-time distribution observed in the pilot/pretest data, responses completed in less than 5 min 30 s or more than 13 min were excluded. The lower cutoff was used to identify responses that were likely completed too quickly to reflect careful reading and valid responding, whereas the upper cutoff was used to screen out cases with unusually long completion times that may have reflected interruption, disengagement, or noncontinuous completion. This step excluded 19 responses. Second, 21 responses showing logical inconsistencies on reverse-scored items were removed. Third, 16 responses failing embedded attention-check items were excluded. These exclusion counts reflect the sequential screening procedure. After screening, 404 valid questionnaires remained, yielding a valid response rate of 88.0%.

Of the valid sample, 199 participants were male (49.3%) and 205 were female (50.7%). The mean age was M = 21.46 years (SD = 1.42). Year of study was distributed as follows: third year, n = 158 (39.1%); second year, n = 146 (36.1%); fourth year, n = 66 (16.3%); first year, n = 34 (8.4%). By major, 247 participants were enrolled in science and engineering programs (61.1%), 141 in humanities and social sciences (34.9%), and 16 in other fields (4.0%).

### 2.2. Measures

#### 2.2.1. Negative Legal Emotion Subscale of the College Students’ Legal Emotion Scale

Negative legal emotion was assessed using the Negative Legal Emotion subscale of the Legal Emotion Assessment Scale for University Students developed by Xu and Yan (2022). This subscale consists of 16 items measuring three dimensions: legal disappointment, legal contempt, and legal aversion. All items were rated on a 5-point Likert scale ranging from 1 (strongly disagree) to 5 (strongly agree), with higher total scores indicating higher levels of negative legal emotion. Example items include “The coercive nature of the law makes me dislike it” and “I dislike judicial institutions such as courts.” In the present study, Cronbach’s alpha was 0.944 for the total scale and 0.831, 0.888, and 0.909 for the three subscales, respectively, indicating good internal consistency.

#### 2.2.2. Prosocial Behavior

Prosocial tendencies were measured using the Chinese version of the Prosocial Tendencies Measure, originally developed by Carlo and Randall (2002) and revised by Kou and Zhang (2006). The scale contains 23 items and assesses six dimensions: anonymous, public, compliant, altruistic, dire, and emotional prosocial tendencies. Responses were made on a 5-point Likert scale ranging from 1 (not at all like me) to 5 (very much like me), with higher scores indicating stronger prosocial tendencies. A sample item is “I feel good when I can comfort someone who is emotionally upset.” In this study, Cronbach’s alpha was 0.967 for the total scale and 0.899, 0.875, 0.859, 0.835, 0.823, and 0.847 for the six subscales, respectively.

#### 2.2.3. Peer Attachment

Peer attachment was assessed using the Inventory of Peer Attachment, originally developed by Armsden and Greenberg (1987) and adapted into Chinese by Zhang Yingli and colleagues. The scale includes 25 items measuring trust, communication, and alienation. Item 5 is reverse-coded. All items were rated on a 5-point Likert scale from 1 (strongly disagree) to 5 (strongly agree). Following Armsden and Greenberg’s (1987) original scoring procedure, an overall peer attachment quality score was calculated by summing the trust and communication scores and subtracting the alienation score, with higher scores indicating stronger peer attachment. A sample item is “I like to ask my friends for their opinions about things that concern me.”

In the present study, this overall peer attachment quality score was used in all main analyses. Cronbach’s alpha was 0.865 for the full scale and 0.903, 0.915, and 0.637 for the trust, communication, and alienation subscales, respectively. Although the alienation subscale showed lower internal consistency than the other two subscales, it was not used as an independent indicator in the mediation analyses. Therefore, the main analyses were based on the overall peer attachment quality score, which showed acceptable reliability in the present sample.

#### 2.2.4. Social Exclusion Experience

Social exclusion was measured using the Social Exclusion Scale developed by Gilman et al. (2013) and revised into Chinese by Zhang Denghao and colleagues. The scale consists of 8 items and includes two dimensions: rejection and neglect. Items were rated on a 7-point Likert scale ranging from 1 (never) to 7 (always), with higher scores indicating stronger experiences of social exclusion. A sample item is “Others usually ignore me as if I were invisible.” In this study, Cronbach’s alpha was 0.948 for the total scale and 0.881 and 0.912 for the two subscales, respectively.

### 2.3. Procedure and Data Analysis

Following approval by the Ethics Review Committee of Wenzhou University and informed consent from all participants, data were collected using an online questionnaire administered in classroom settings at five universities in mainland China: Ludong University, Yantai Institute of Technology, Northwest University, Changzhou Institute of Technology, and Wenzhou University. Students completed the survey individually on their own devices during class time under group administration. Participation was voluntary, and respondents received a randomly assigned monetary incentive of 7–10 RMB upon completion. To reduce common method bias, reverse-coded items were included and responses were collected anonymously. Data quality was carefully screened, and responses with abnormal completion times, logical inconsistencies, or invalid answers were excluded from the final dataset.

All analyses were conducted using SPSS 27.0. Descriptive statistics, independent-samples *t* tests, one-way ANOVAs, and correlation analyses were first used to examine the variables. To assess potential common method bias, both Harman’s single-factor test and a single-factor confirmatory factor analysis were conducted. Harman’s test showed that 11 factors with eigenvalues greater than 1 were extracted, and the largest factor accounted for 35.03% of the total variance, below the commonly used 40% threshold. In addition, the single-factor confirmatory factor analysis including all 80 items showed poor fit, χ^2^(3080) = 13,786.28, *p* < 0.001, CFI = 0.533, TLI = 0.521, RMSEA = 0.093, and SRMR = 0.111, suggesting that a single common factor could not adequately explain the data. Thus, although common method bias cannot be entirely excluded, it was unlikely to represent a serious concern in the present study.

For subsequent analyses, composite scores were calculated for each construct. Although some scales included multiple subdimensions, previous validation studies have supported their use as indicators of broader constructs. Consistent with common practice in Hayes’s PROCESS analyses, these composite scores were used in the mediation and moderation models.

The mediation and moderation effects were tested using the PROCESS macro for SPSS.

## 3. Results

### 3.1. Demographic Differences in Key Variables

To examine demographic differences in the study variables, independent-samples *t* tests and one-way analyses of variance (ANOVAs) were conducted as appropriate. For two-group comparisons, Levene’s test was first used to assess the homogeneity of variances. When the homogeneity assumption was violated, Welch’s *t* test was used; therefore, some *t* tests are reported with fractional degrees of freedom.

According to the independent-samples *t* tests, a significant gender difference was found in perceived social exclusion, t(389.87) = 2.00, *p* = 0.046, with males (M = 21.18, SD = 10.60) scoring significantly higher than females (M = 19.21, SD = 9.14). No significant gender differences were observed for total peer attachment, t(390.26) = −1.51, *p* = 0.133, prosocial tendency, t(382.79) = −1.45, *p* = 0.148, or negative legal emotion, t(368.91) = 1.88, *p* = 0.061.

Regarding academic major, students in the humanities and social sciences did not differ significantly from those in science and engineering on perceived social exclusion, t(320.59) = −1.01, *p* = 0.314, total peer attachment, t(285.88) = 1.54, *p* = 0.125, prosocial tendency, t(261.38) = 0.23, *p* = 0.820, or negative legal emotion, t(338.69) = −1.36, *p* = 0.175. Other major categories were excluded from the analysis because their sample sizes were too small to support reliable statistical inference.

One-way analyses of variance were conducted to examine grade-level differences in the study variables. The results showed a significant grade effect for negative legal emotion, F(3, 400) = 2.88, *p* = 0.036. Post hoc comparisons using the LSD test indicated that fourth-year students reported significantly lower negative legal emotion than first-year students (MD = −0.503, *p* = 0.017), second-year students (MD = −0.360, *p* = 0.015), and third-year students (MD = −0.370, *p* = 0.011), whereas no significant differences were found among first-, second-, and third-year students (ps > 0.05). No significant grade differences were found for total peer attachment, F(3, 400) = 1.482, *p* = 0.219, perceived social exclusion, F(3, 400) = 0.282, *p* = 0.838, or prosocial tendency, F(3, 400) = 1.107, *p* = 0.346. Overall, only negative legal emotion varied significantly across grade levels, with fourth-year students scoring lower than students in the other three grades.

One-way ANOVA examining paternal educational level showed a significant group difference only for total peer attachment, F(4, 399) = 2.80, *p* = 0.026. LSD post hoc comparisons indicated that students whose fathers had a university education reported significantly higher peer attachment than those whose fathers had a junior high school education (MD = 0.294, *p* = 0.028) or primary school education or below (MD = 0.672, *p* = 0.006). In addition, students whose fathers had a senior high school education scored significantly higher on peer attachment than those whose fathers had a primary school education or below (MD = 0.566, *p* = 0.019). In general, higher paternal educational attainment was associated with higher levels of peer attachment. No significant effects of paternal education were found for prosocial tendency, F(4, 399) = 1.85, *p* = 0.118, perceived social exclusion, F(4, 399) = 0.81, *p* = 0.522, or negative legal emotion, F(4, 399) = 1.07, *p* = 0.373.

Analyses of maternal educational level showed significant group differences in prosocial tendency, F(4, 399) = 3.84, *p* = 0.004, total peer attachment, F(4, 399) = 6.40, *p* < 0.001, and negative legal emotion, F(4, 399) = 6.46, *p* < 0.001, whereas perceived social exclusion did not differ significantly across groups, F(4, 399) = 1.31, *p* = 0.264. LSD post hoc comparisons showed that, for negative legal emotion, students whose mothers had a primary school education or below scored significantly higher than those whose mothers had a junior high school, senior high school, or university education (*p*s < 0.001). For peer attachment, students whose mothers had a primary school education or below scored significantly lower than those whose mothers had a junior high school, senior high school, or university education (*p*s < 0.001). For prosocial tendency, students whose mothers had a primary school education or below also scored significantly lower than those whose mothers had a junior high school, senior high school, or university education (*p* = 0.001). No other pairwise differences were significant (*p*s > 0.05). Overall, higher maternal educational attainment was associated with lower negative legal emotion and higher peer attachment and prosocial tendency.

Based on these demographic difference analyses, only demographic variables showing significant group differences were retained as control variables in the subsequent regression models to improve the accuracy of parameter estimation.

### 3.2. Descriptive Statistics and Correlational Analyses of Key Variables

Descriptive statistics and Pearson correlation analyses among the primary study variables revealed several significant associations. Social exclusion was significantly and negatively correlated with prosocial behavior, r = −0.44, *p* < 0.001, and overall peer attachment, r = −0.57, *p* < 0.001, and was significantly and positively correlated with negative legal emotion, r = 0.48, *p* < 0.001. Prosocial behavior was positively associated with overall peer attachment, r = 0.67, *p* < 0.001, and negatively associated with negative legal emotion, r = −0.42, *p* < 0.001. In addition, overall peer attachment showed a significant negative correlation with negative legal emotion, r = −0.58, *p* < 0.001. See Table 1.

### 3.3. Results of the Mediation and Moderation Analyses

Hayes’s (2018) PROCESS macro (Model 7) was used to examine the mediating role of peer attachment in the association between negative legal emotion and prosocial behavior, as well as the moderating effect of social exclusion on the first stage of this mediation pathway, while controlling for gender, grade, and parental educational attainment (Hayes, 2018).

In the regression model predicting peer attachment (Model 1), negative legal emotion significantly and negatively predicted peer attachment (β = −0.502, *p* < 0.001), and social exclusion also significantly and negatively predicted peer attachment (β = −0.408, *p* < 0.001). In addition, the interaction between negative legal emotion and social exclusion significantly and positively predicted peer attachment (β = 0.181, *p* < 0.001), indicating that social exclusion moderated the association between negative legal emotion and peer attachment. This model accounted for 50.20% of the variance in peer attachment (R^2^ = 0.502, F(13, 390) = 30.25, *p* < 0.001).

In the regression model predicting prosocial behavior (Model 2), the direct effect of negative legal emotion on prosocial behavior was not significant (β = −0.037, *p* = 0.429), whereas peer attachment significantly and positively predicted prosocial behavior (β = 0.636, *p* < 0.001). This model explained 45.37% of the variance in prosocial behavior (R^2^ = 0.454, F(12, 391) = 27.06, *p* < 0.001). Detailed results are presented in Table 2.

**Figure 2:**
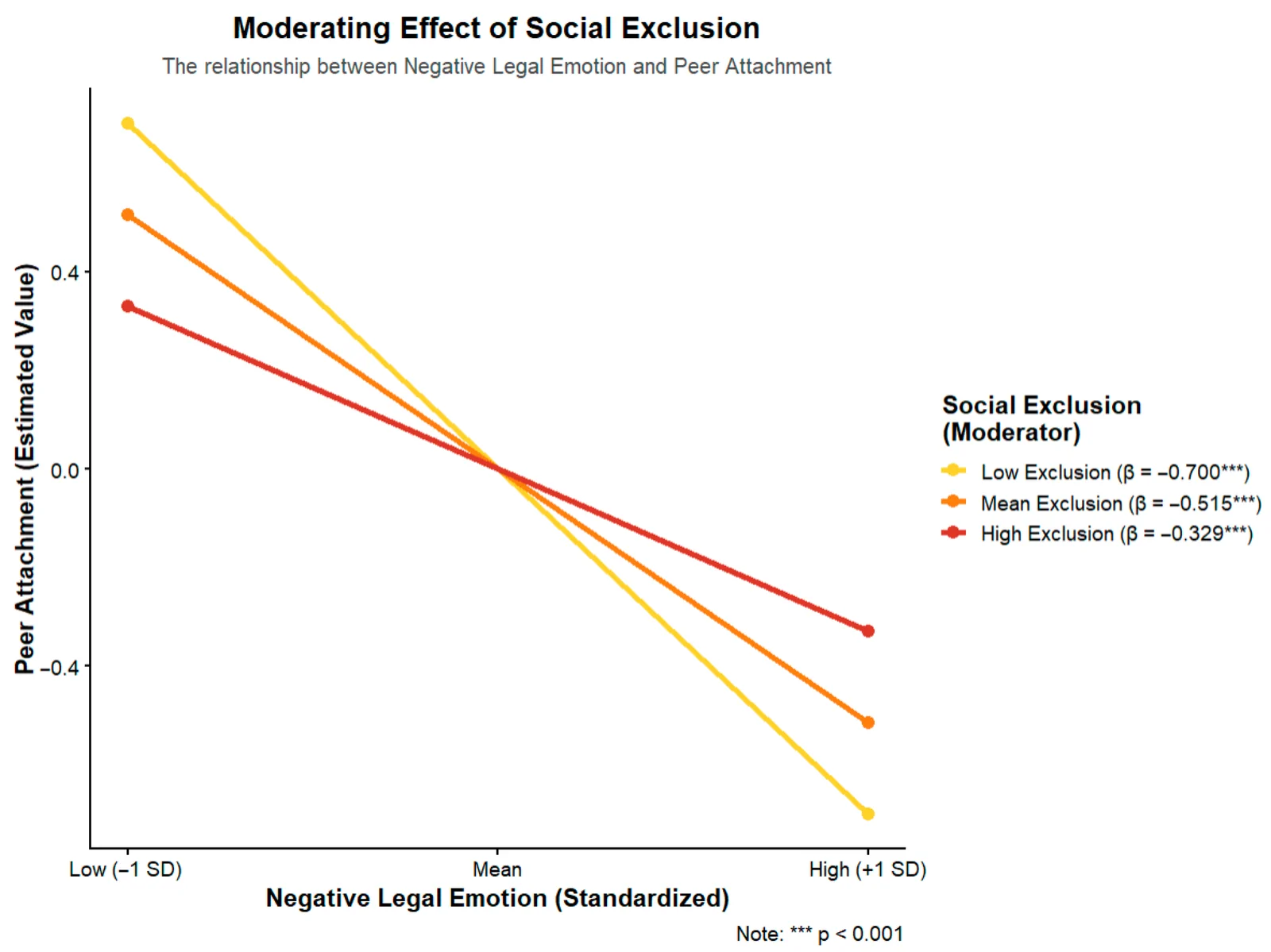
The Moderating Effect of Social Exclusion on the Relationship Between Negative Legal Emotion and Peer Attachment.

The simple slope analysis showed that the negative predictive effect of negative legal emotion on peer attachment became progressively weaker as levels of social exclusion increased. Specifically, at low social exclusion (−1 SD), the effect was β = −0.683, *p* < 0.001; at the mean level, β = −0.502, *p* < 0.001; and at high social exclusion (+1 SD), β = −0.321, *p* < 0.001 (see Figure 2).

The Johnson–Neyman technique was further used to examine the moderating effect of social exclusion. The results showed that when the standardized score of social exclusion was below 2.0815, the negative effect of negative legal emotion on peer attachment was significant (*p* < 0.05), and this range covered 95.79% of the sample. As the level of social exclusion increased, this negative effect gradually weakened: when the social exclusion score increased from −1.2271 to 1.9161, the effect size decreased from −0.7235 (*p* < 0.001) to −0.1560 (*p* = 0.0094). When the social exclusion score reached 2.0815, the effect became marginally significant (β = −0.1261, *p* = 0.0500); beyond this value, the effect was no longer significant (*p* > 0.05). These findings further support the buffering effect of social exclusion, indicating that the higher the level of social exclusion, the weaker the negative impact of negative legal emotion on peer attachment.

Bootstrap analyses further showed that the indirect effect of negative legal emotion on prosocial behavior through peer attachment was significant at all levels of social exclusion, but the magnitude of the effect decreased as social exclusion increased. At low social exclusion, the indirect effect was −0.434, 95% CI [−0.590, −0.309]; at the mean level, the indirect effect was −0.319, 95% CI [−0.424, −0.235]; and at high social exclusion, the indirect effect was −0.204, 95% CI [−0.280, −0.141]. The index of moderated mediation was significant (Index = 0.115, 95% CI [0.060, 0.178]), indicating that the higher the level of social exclusion, the weaker the indirect pathway through which negative legal emotion reduced prosocial behavior by undermining peer attachment (see Table 3).

## 4. Discussion

The present findings suggest that negative legal emotion, peer attachment, prosocial behavior, and social exclusion are linked in a conditional relational pattern. Negative legal emotion was negatively associated with prosocial behavior, and this association was statistically accounted for by peer attachment. This pattern is consistent with the view that legal–emotional orientations are closely related to social functioning and that peer relationships constitute an important interpersonal context for behavioral adjustment (He et al., 2025). In this sense, the findings extend prior work by suggesting that peer attachment may serve as an important relational correlate linking negative legal emotion with lower prosocial behavior among adolescents and university students.

The role of social exclusion was more complex. At the bivariate level, social exclusion was negatively associated with prosocial behavior, consistent with prior work linking exclusion to negative affect, poorer self-regulation, and lower prosocial responding (Baumeister et al., 2002; Twenge et al., 2007; Williams, 2007). At the same time, within the conditional process model, higher social exclusion was associated with a weaker negative association between negative legal emotion and peer attachment. This pattern is broadly consistent with the multimotive model of interpersonal rejection, which suggests that exclusion may be associated not only with avoidant or antagonistic responses but also with affiliative motives aimed at restoring belonging (Smart Richman & Leary, 2009). Related studies have likewise shown that exclusion may, under some conditions, heighten motivation for reconnection and increase sensitivity to social acceptance cues (Maner et al., 2007; Williams et al., 2000). This interpretation may be especially relevant in Chinese samples. Research with Chinese participants has shown that interdependent self-construal is associated with more adaptive coping following ostracism (Ren et al., 2013), and recent meta-analytic evidence suggests that social exclusion may be followed by stronger prosocial responses in collectivistic cultural contexts (Lin et al., 2025). In addition, evidence from the Chinese context suggests that exclusion is embedded in a relational–cultural environment in which interpersonal ties, social circles, and belonging carry heightened significance (Li & Li, 2025). Accordingly, although social exclusion was associated with lower prosociality overall, it may also be linked to stronger relationship-maintenance or reconnection tendencies in specific interpersonal pathways. This pattern should not be interpreted as indicating that exclusion is beneficial overall. Rather, under higher exclusion, remaining peer bonds may become relatively more salient as a source of belonging, such that the negative association between negative legal emotion and peer attachment appears weaker within this specific pathway. Psychologically, this pattern may reflect a remaining-bond preservation process: when exclusion is higher, available peer ties may be valued more strongly as comparatively scarce sources of belonging, making peer attachment less susceptible to the negative association otherwise linked to negative legal emotion.

Taken together, these findings support a context-dependent interpretation of social exclusion rather than a uniformly detrimental or uniformly compensatory one. As a general interpersonal condition, exclusion was negatively associated with prosocial behavior; however, within the moderated mediation model, it was associated with a weaker negative link between negative legal emotion and peer attachment. Importantly, the present measure captured self-reported social exclusion as an ongoing interpersonal experience rather than experimentally induced momentary ostracism, which may partly explain why the present pattern does not map directly onto classic ostracism findings. More broadly, by examining negative legal emotion, peer attachment, and social exclusion within the same framework, the present study adds to the literature by suggesting that the association between legal–emotional orientations and prosocial behavior may depend not only on peer attachment itself but also on the broader relational climate in which peer bonds are embedded. This compensatory affiliation interpretation may be especially relevant for individuals who continue to value peer belonging and perceive existing peer ties as psychologically available, a possibility that should be examined more directly in future research.

### Theoretical and Practical Implications

The present findings have several implications for legal socialization theory. First, they suggest that legal socialization may be understood not only in terms of legal cognition and institutional evaluation, but also in terms of the affective orientations through which individuals relate to law in everyday life (Fu, 2016; Xu, 2026). In this respect, the findings support a more relational view of legal socialization, in which institution-related emotion is linked to everyday social functioning rather than confined to abstract judgments about institutions. This interpretation is also consistent with broader theoretical work suggesting that legal socialization involves not only compliance-related beliefs, but also the emotional and relational processes through which individuals orient themselves toward law and authority (Trinkner & Tyler, 2016).

Second, the findings contribute to broader discussions of institutional trust and youth social development. Rather than linking negative legal emotion solely to deviance or legitimacy judgments, the present study suggests that institution-related negative emotion may also be associated with peer relational functioning. This does not mean that institutions and peers are equivalent targets. Instead, it suggests that weaker affective endorsement of law may correspond to broader patterns of lower normative trust, reduced social investment, and weaker relational security, which may become particularly salient in peer contexts during emerging adulthood. For university students, peer relationships represent a central microsocial context for belonging, adjustment, and everyday social behavior (Buote et al., 2007; Calderon Leon et al., 2024; Maunder, 2018; Song et al., 2022; Swenson et al., 2008). The present findings therefore place legal socialization more clearly within the developmental context of young adulthood.

The findings also have practical implications. Efforts to promote prosocial behavior among university students may benefit from focusing not only on legal knowledge and rule awareness, but also on students’ emotional orientations toward law and the peer relational contexts in which prosocial tendencies are expressed. Peer belonging, supportive peer climates, and institution-related emotional experiences may all be relevant to student adjustment and prosocial development. More broadly, the study may contribute to interdisciplinary dialogue between psychology and sociology by illustrating how institutional and interpersonal domains may intersect in the development of prosocial tendencies (Tyler, 2006). Although the present study did not directly examine overseas Chinese students, the findings may have limited tentative relevance for this population. In contexts of cultural transition and adaptation, belonging, exclusion, and peer support may be especially important for social adjustment. In some cases, overseas Chinese students who express attachment to their home-country identity may encounter misunderstanding or social exclusion, which may further underscore the importance of supportive peer relationships and inclusive campus climates. From this perspective, these relational contexts may help foster prosocial development.

More broadly, the present findings show both continuity with and specificity relative to prior research. They align with earlier work highlighting the importance of peer relationships and social exclusion in youth social functioning, while extending this literature by linking institution-related negative emotion to peer relational processes in a university student sample. This specificity may be particularly relevant in the present Chinese context, where relational interdependence and norm-related meanings of belonging may shape these associations.

## 5. Conclusions, Limitations, and Future Directions

The present study suggests that negative legal emotion, peer attachment, prosocial behavior, and social exclusion are linked in a conditional relational pattern among university students. Negative legal emotion was negatively associated with prosocial behavior, and this association was statistically accounted for by peer attachment. In addition, the indirect association via peer attachment varied across levels of social exclusion. These findings highlight the relevance of peer relationships in the context of legal socialization and suggest that legal–emotional orientations may be associated with prosocial behavior partly through peer relational functioning.

Several limitations should be noted. First, the cross-sectional design does not permit strong temporal or directional conclusions, and the findings should therefore be interpreted as conditional associations. Second, the sample consisted of Chinese university students, so the observed pattern may be shaped by cultural factors such as collectivistic orientation, relational interdependence, norm compliance, and institutional trust, which may limit generalizability. Third, all variables were assessed using self-report measures, which may introduce shared-method, social desirability, and subjective interpretation biases. Accordingly, common method variance cannot be fully ruled out. Fourth, social exclusion was assessed as self-reported ongoing exclusion rather than experimentally induced momentary ostracism, which may partly explain differences from classic ostracism findings. Finally, although social exclusion was modeled as a moderator, alternative model structures are also plausible, and relevant individual differences were not directly assessed.

Future research would benefit from longitudinal, experimental, multi-method, and model-comparison approaches. It would also be valuable to test whether the present pattern generalizes across different age groups, cultural contexts, and forms of social exclusion, and whether factors such as collectivism, interdependent self-construal, and institutional trust function as meaningful boundary conditions.

## Figures and Tables

**Table 1:** Descriptive Statistics and Correlations Among Study Variables.

Variables	1	2	3	4
1. Negative Legal Emotion	1			
2. Social Exclusion	0.48 ***	1		
3. Prosocial Behavior	−0.42 ***	−0.44 ***	1	
4. Peer Attachment	−0.58 ***	−0.57 ***	0.67 ***	1
*M ± SD*	1.49 ± 0.57	20.18 ± 9.93	105.78 ± 18.12	55.86 ± 14.34

*** *p* < 0.001.

**Table 2:** Regression Analysis Results of the Moderated Mediation Model (N = 404).

Variables	Model 1 (Dependent Variable: Peer Attachment)			Model 2 (Dependent Variable: Prosocial Behavior)		
	*β*	*SE*	*t*	*β*	*SE*	*t*
Constant	−0.434	0.214	−2.027 *	−0.034	0.224	−0.154
NLE	−0.502	0.051	−9.865 ***	−0.037	0.047	−0.792
PA				0.636	0.047	13.634 ***
SE	−0.408	0.042	−9.823 ***			
NLE × SE	0.181	0.037	4.940 ***			
Gender	−0.021	0.072	−0.29	−0.033	0.076	−0.43
Grade_S	0.13	0.139	0.935	−0.054	0.145	−0.37
Grade_J	0.038	0.137	0.28	0.077	0.143	0.537
Grade_SE	0.029	0.155	0.19	0.019	0.162	0.117
FEM_M	0.036	0.178	0.201	−0.165	0.185	−0.893
FEM_H	0.021	0.187	0.111	−0.036	0.194	−0.185
FEM_C	0.06	0.205	0.295	−0.006	0.211	−0.027
ME_M	0.272	0.153	1.774	0.143	0.16	0.899
ME_H	0.27	0.167	1.617	0.133	0.173	0.768
ME_C	0.296	0.19	1.555	0.001	0.197	0.003
R2	0.502			0.454		
F	F(13, 390) = 30.25 ***			F(12, 391) = 27.06 ***		

NLE = Negative Legal Emotion; SE = Social Exclusion; PA = Peer Attachment; Grade_S = Grade_Sophomore; Grade_J = Grade_Junior; Grade_SE = Grade_Senior; FEM_M = Father Education_Middle; FEM_H = School Father Education_High School; FEM_C = Father Education_College or Above; ME_M = Mother Education_Middle School; ME_H = Mother Education_High School; ME_C = Mother Education_College or Above. Grade was dummy-coded with freshman as the reference group. Parental education was dummy-coded with primary school or below as the reference group. * *p* < 0.05, *** *p* < 0.001.

**Table 3:** Conditional Indirect Effects and Index of Moderated Mediation.

Moderator Level Indirect Effect	Moderator Level Indirect Effect	Boot SE	BootLLCI	BootULCI
Low Social Exclusion (−1 SD)	−0.434	0.071	−0.590	−0.309
Mean Level	−0.319	0.048	−0.424	−0.235
High Social Exclusion (+1 SD)	−0.204	0.036	−0.280	−0.141
Index of Moderated Mediation	Index	Boot SE	BootLLCI	BootULCI
Social Exclusion	0.115	0.03	0.06	0.178

Bootstrap resampling = 5000. Indirect effect represents the conditional indirect effect of negative legal emotion on prosocial behavior via peer attachment.

## Data Availability

The raw data supporting the conclusions of this article will be made available by the authors on request.
